# Recent developments in mesoporous polydopamine-derived nanoplatforms for cancer theranostics

**DOI:** 10.1186/s12951-021-01131-9

**Published:** 2021-11-24

**Authors:** Menglu Zhu, Yi Shi, Yifan Shan, Junyan Guo, Xuelong Song, Yuhua Wu, Miaolian Wu, Yan Lu, Wei Chen, Xiaoling Xu, Longguang Tang

**Affiliations:** 1 The Fourth Affiliated Hospital, Zhejiang University School of Medicine, 322000 Yiwu, Zhejiang People’s Republic of China; 2 Longhua Hospital, Shanghai University of Traditional Chinese Medicine, 200032 Shanghai, People’s Republic of China; 3 Shulan International Medical College, Zhejiang Shuren University, 310004 Hangzhou, Zhejiang People’s Republic of China; 4 International Institutes of Medicine, The Fourth Affiliated Hospital, Zhejiang University School of Medicine, 322000 Yiwu, Zhejiang People’s Republic of China

**Keywords:** MPDA, Nanodrug delivery systems, Photothermal therapy, Cancer theranostics, Immunotherapy

## Abstract

Polydopamine (PDA), which is derived from marine mussels, has excellent potential in early diagnosis of diseases and targeted drug delivery owing to its good biocompatibility, biodegradability, and photothermal conversion. However, when used as a solid nanoparticle, the application of traditional PDA is restricted because of the low drug-loading and encapsulation efficiencies of hydrophobic drugs. Nevertheless, the emergence of mesoporous materials broaden our horizon. Mesoporous polydopamine (MPDA) has the characteristics of a porous structure, simple preparation process, low cost, high specific surface area, high light-to-heat conversion efficiency, and excellent biocompatibility, and therefore has gained considerable interest. This review provides an overview of the preparation methods and the latest applications of MPDA-based nanodrug delivery systems (chemotherapy combined with radiotherapy, photothermal therapy combined with chemotherapy, photothermal therapy combined with immunotherapy, photothermal therapy combined with photodynamic/chemodynamic therapy, and cancer theranostics). This review is expected to shed light on the multi-strategy antitumor therapy applications of MPDA-based nanodrug delivery systems.

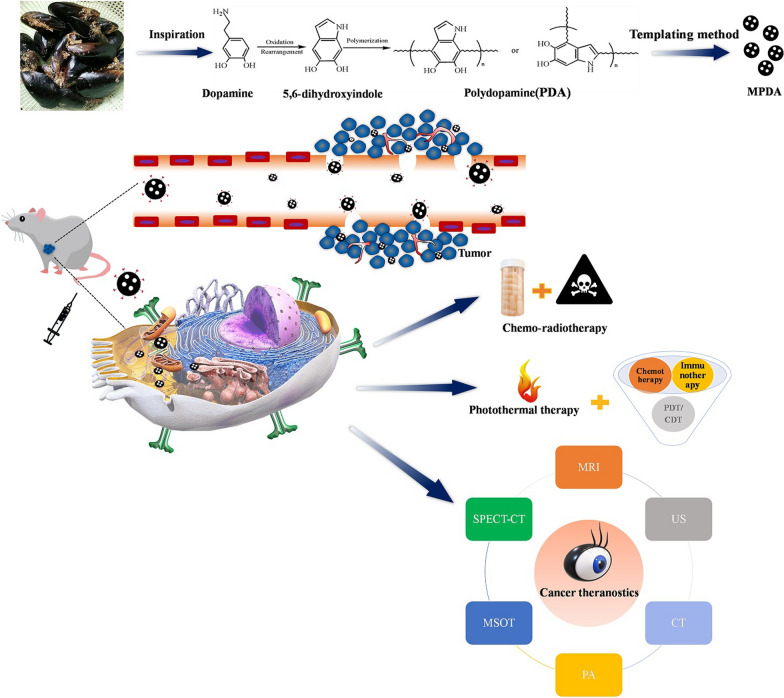

## Introduction

Cancer is the second-leading cause of mortality worldwide, with increasing number of deaths and incidences. According to the International Agency for Research on Cancer statistics, there were 19.3 million new cancer cases and nearly 10 million deaths in 2020 globally. By 2040, the number of new cancer cases is predicted to reach 28.4 million [1]. However, the current state of cancer prevention and treatment is still concerning. Consequently, cancer has become a crucial problem that endangers human life and health, and imposes a substantial economic burden on society and individuals.

Currently available cancer treatments include surgery, radiotherapy, chemotherapy, and immunotherapy [2–4]. Although these treatments have achieved promising outcomes, they still have several limitations. For example, surgical treatment is mostly used to remove early solid tumors that have not spread and metastasized. Although radiotherapy is relatively effective against some tumors, it has limited dosage regimen and can cause damage to normal human tissues. Moreover, muscle sequelae and nerve damage can occur after radiotherapy, and the recovery is challenging. Chemotherapy, as a systemic treatment for cancer therapy, employs cytotoxic drugs that directly kill tumor cells; however, although it has a good curative effect on primary, metastatic, and subclinical metastatic cancers, chemotherapy frequently causes severe toxic and side effects, resulting in increased drug tolerance (even in multiple chemotherapies) and eventually leading to treatment failure [5, 6]. Despite the advancement of immunotherapy in cancer treatment, its curative effect remains unsatisfactory owing to the complex tumor immune microenvironment. Therefore, immunotherapy is now often used in combination with surgery, radiotherapy, and chemotherapy [7].

Recently, nanodrug delivery system-mediated high-end formulations have been used to significantly improve drug distribution in the body, thus enhancing the curative benefits and reducing toxic and side effects. However, they are still restricted by low drug-loading efficiency and single treatment mode, resulting in unsatisfactory curative effect and limited theranostic applications. Polydopamine (PDA) has emerged as a promising nanomaterial to overcome these difficulties.

Lee et al. [8] reported a material surface modification method based on mussel bionics. This method was based on an adhesion protein that can attach to the surface of almost all materials, including inorganic and organic materials. The adhesion ability resulted from their large amount of levodopa structure (L-3,4 dihydroxyphenylalanine, L-dopa)-based mucus proteins. Together, the amino and catechol groups of L-dopa form strong covalent and non-public bonds on the surface of the substrate, promoting adhesion. PDA is a biomimetic polymer obtained through polymerization of dopamine monomer under alkaline conditions. It possesses unique physical and chemical features, including metal ion chelation, photothermal conversion, near-infrared (NIR) responsiveness, high biocompatibility and biodegradability, accessible surface modification, and effective drug-loading capacity. These features are responsible for the improved performance of PDA compared with other nanomaterials. For this reason, PDA has recently been widely applied in tumor nanodrug delivery systems [9–11].

Chemotherapeutic drugs are mainly loaded onto PDA nanoparticles through physical adsorption mediated by π-π stacking. However, traditional PDA nanoparticles are prepared through simple self-polymerization in alkaline solution without templates, and the nanoparticles obtained via this preparation method display limited specific surface area. Moreover, the loaded drugs are easily separated under complex physiological conditions. Therefore, it is urgent to develop PDA nanoparticles with improved structures that allow enhanced drug-loading efficiency and on-demand drug release.

Despite these challenges, researchers continue to innovate and optimize the preparation methods to achieve higher drug-loading and tumor suppression efficiencies compared to a solid PDA nanoparticle. With the rapid development of nanotechnology, porous materials have become a research hotspot owing to their unique structure as well as good physical and chemical properties [12–14]. For example, mesoporous silicon has attracted wide attention because of its uniform and adjustable mesoporous pore size, stable framework structure, large specific surface area, and modifiable inner surface. In recent years, PDA-modified mesoporous silica has been used in tumor treatment, as shown in Table 1. However, the practical applications of mesoporous silicon are limited by the pore size and structure [47]. They cannot achieve on-demand loading efficiency, especially when delivering substrates of specific sizes, such as biological macromolecules (e.g., nucleotides and proteins) [48].

Nanobottles have also received considerable attention because they can quickly load chemical reagents, biological effectors, therapeutic/diagnostic agents, and nano-scale objects. They have been applied in areas such as catalysis, pollutant separation, controlled release, and drug delivery. The high loading capacity of nanobottles ensure the minimal use of carrier materials, thus reducing their potential toxicity during treatment. Furthermore, the opening of nanobottles allows the loading or release of all kinds of loads, regardless of their size or hydrophobicity. The opening can also be designed with smart functions to realize the stimulus-response release of payload. However, the depth and size of a pore may affect complete drug release [49, 50]. As nanobottles have only one hole with a depth of up to approximately 500 nm, they may not release a drug completely [51].

**Scheme 1:**
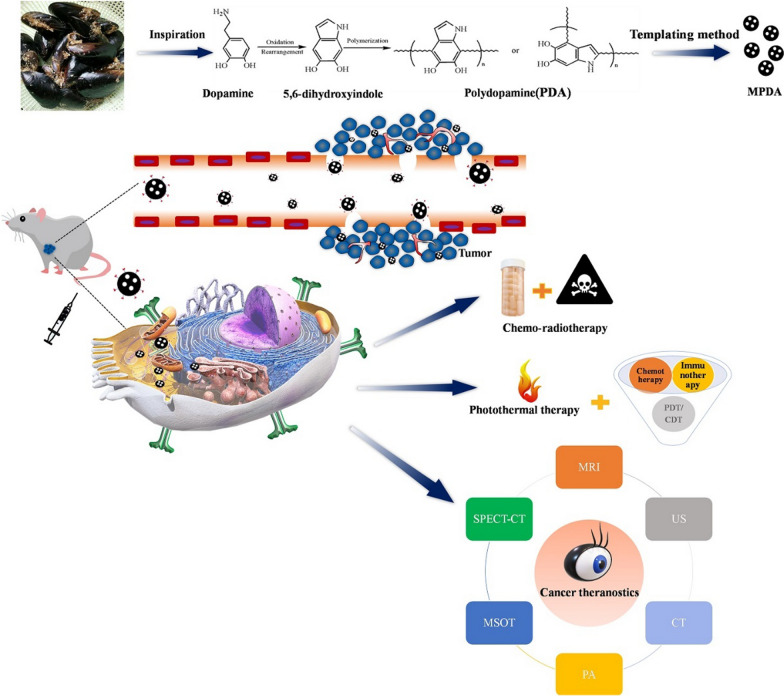
Antitumor and imaging applications of mesoporous polydopamine-derived nanoplatforms

To overcome these challenges, Tang et al. developed a preparation procedure for mesoporous carbon material using high-molecular-weight block copolymers as templates and successfully synthesized mesoporous polydopamine (MPDA), which enhanced the research potential of PDA [52]. Compared with that of nanobottles, the pores of MPDA are mesopores having a depth and size of 2–50 nm. This pore size and depth allow on-demand drug loading and complete drug release. Moreover, previous reports revealed that nanocarriers with a particle size of < 200 nm are expected to accumulate in the tumor site through the enhanced permeability and retention effect [53]. Hence, MPDA-based nanoplatforms are promising in targeted drug delivery for cancer treatment. New advances in MPDA have provided a new approach to achieve high drug-loading capacity, multimodal anticancer treatment, and visual therapy. Therefore, we herein review the preparation methods and antitumor applications of MPDA (Scheme 1).

**Table 1:** Polydopamine-modified mesoporous silica applied in tumor treatment

Formulation	Drug	Cancer	Therapy	Imaging	Application	Refs.
Nanoparticles	USIONPs	4T1	PTT- RT	MRI, PA	Cancer theranostics	[15]
Nanoparticles	Cur/ Silver	HeLa, A549/TAX	Chemotherapy		Cancer therapy	[16]
Nanoparticles	DOX	A549, A549/MDR	Chemotherapy		Multidrug resistance therapy	[17]
Nanoparticles	DOX	Hela	Chemotherapy		Targeted therapy	[18]
Nanoparticles	DOX	HT-1376	Chemotherapy		Targeted therapy	[19]
Nanoparticles	CQ	HepG-2	PTT	PA	Cancer therapy	[20]
Nanoparticles	DOX	4T1	Chemo-photothermal therapy		Cancer therapy	[21]
Nanoparticles	DES	HeLa	Chemotherapy		Cancer therapy	[22]
Nanoparticles	DM1	SW480	Chemotherapy		Targeted therapy	[23]
Nanoparticles	DOX, QUR	HCT-8, HCT-8/TAX	Chemotherapy		Multidrug resistance therapy	[24]
Nanoparticles	DOX, ICG	4 T1, MCF-7	Chemo-photothermal therapy	MRI, PA	Cancer theranostics	[25]
Nanoparticles	DOX	HepG2	Chemo-photothermal therapy	PA	Cancer theranostics	[26]
Nanoparticles	PTX	4T1	Chemotherapy		Cancer therapy	[27]
Nanoparticles	DOX	HeLa	Chemo-photothermal therapy		Targeted therapy	[28]
Nanoparticles	PFH/ICG	MCF-7	PTT/PDT	US/NIRF	Cancer theranostics	[29]
Nanoparticles	DOX	MHCC97L, MHCC97H	Chemo-photothermal therapy		Cancer therapy	[30]
Nanoparticles	DOX	MDA-MB-231	Chemo- photothermal therapy		Cancer therapy	[31]
Nanoparticles	DOX, PFP	MDA-MB-231	Chemo- photothermal therapy	US	Cancer theranostics	[32]
Nanoparticles	DOX	MCF-7	Chemotherapy	US	Targeted therapy	[33]
Nanoparticles	ABC, DOX, ICG	CT26	Chemo-photothermal therapy		Cancer therapy	[34]
Nanoparticles	CX-5461	HeLa			Targeted therapy	[35]
Nanoparticles	DOX	HepG2	Chemo-photothermal therapy/ Starvation therapy/Oxidative therapy		Targeted therapy	[36]
Nanoparticles	anti-miR-155	SW480	Gene therapy		Targeted therapy	[37]
Nanoparticles	TMZ	C6			Targeted therapy	[38]
Nanoparticles	PFH	4T1	PTT	PA/US/CT	Cancer theranostics	[39]
Nanoparticles	DOX	HeLa	Chemo-photothermal therapy		Cancer therapy	[40]
Nanoparticles	DOX, CUR	MCF-7/ADR	Chemotherapy		Multidrug resistance therapy	[41]
Nanoparticles	DOX	HeLa	Chemo-photothermal therapy		Targeted therapy	[42]
Nanoparticles	Cisplatin	HeLa	PTT		Cancer therapy	[43]
Nanoparticles	DOX,	HepG2			Cancer therapy	[44]
Nanoparticles	DOX, ICG, ABC	CT26	PTT-PDT- Chemotherapy		Targeted therapy	[45]
Nanoparticles	Gardiquimod	B16-F10	Photothermal-immunotherapy		Immunotherapy	[46]

## Mesoporous polydopamine preparation

The templating method is utilized to synthesize mesoporous carbon materials with ordered mesoporous structures. Owing to its large pore size and controllable distribution, it is widely used in various fields. This method can be divided into two types, hard (nanocasting) [54] and soft templating, according to the characteristics of the template itself and the difference in domain limitation ability. Generally, template synthesis entails three steps: preparation, template-directed synthesis, and removal. The primary process of hard templating is selection and removal, and the main process of soft templating is self-assembly and carbonization [55]. Template synthesis of nanomaterials has the following advantages: (1) it allows the precise control of size, shape, structure, and other properties; (2) it enables nanomaterial synthesis and assembly integration, and solves nanomaterial dispersion issues; (3) the preparation process is simple and suitable for industrial production.

### Hard templating

The hard templating method, also called nanocasting, involves covalent bonds to maintain a specific shape. It mostly refers to the rigid templates maintained by covalent bonds, such as high-molecular-weight polymers with different spatial structures, anodic aluminum oxide membranes, porous silicon, metal template natural high-molecular-weight materials, molecular sieves, colloidal crystals, and carbon nanotubes [56–59]. The hard template synthesis method is based on the deposition of a target material into the narrow space of the template, resulting in reverse replication of the mold. As the template must be wetted by the precursor molecules, it can be affected by the random distribution of carbon components, pore-clogging, phase separation, and disordered pore structure. The hard template mainly provides a mesoscopic confined space as a “filler” of the space. The key to this controlled synthesis in the rigid framework is how to “fill” the entire mesoporous channels of the hard template by “relying on” the capillary force. The morphology of the porous carbon material prepared via the hard templating method is mainly determined by the template matrix. Studies on the hard templating method mainly investigated templates with different structures. Hard templates have high stability and can strictly control the size and morphology of nanomaterials. However, their structure is relatively simple, and thus the morphology of nanomaterials prepared using hard templates usually does not significantly change. In this method, a hard template must be prepared in the early stage and then removed in the later stage; thus, the process is complicated, time-consuming, and low in yield. For example, the hard templating method using SiO_2_ as the template mainly controls the size of SiO_2_ spheres and selects appropriate precursors to realize the adjustment of the mesoporous carbon spheres pores [60]. Zhao et al. [61] regarded CdS NRs (CdS nanorods) as sulfur sources and hard templates, and applied Prussian blue analogs (PBA) anchored on PDA-coated CdS nanorods as precursors to prepare ultrafine Co4S3(cobalt sulfides) nanoparticles supported on N, S-codoped CNTs (Co_4_S_3_@N, S-CNT). Following pyrolysis and wet etching with HCl, Co_4_S_3_ @N, S-CNT-800 was obtained. This method can be used to obtain macroporous carbon nanospheres, but the synthesis process is complicated and time-consuming, and the hard template must be removed in the later stage. Moreover, the strong corrosive solution used in the template synthesis process is dangerous and not suitable for large-scale production.

### Soft templating

**Fig. 1:**
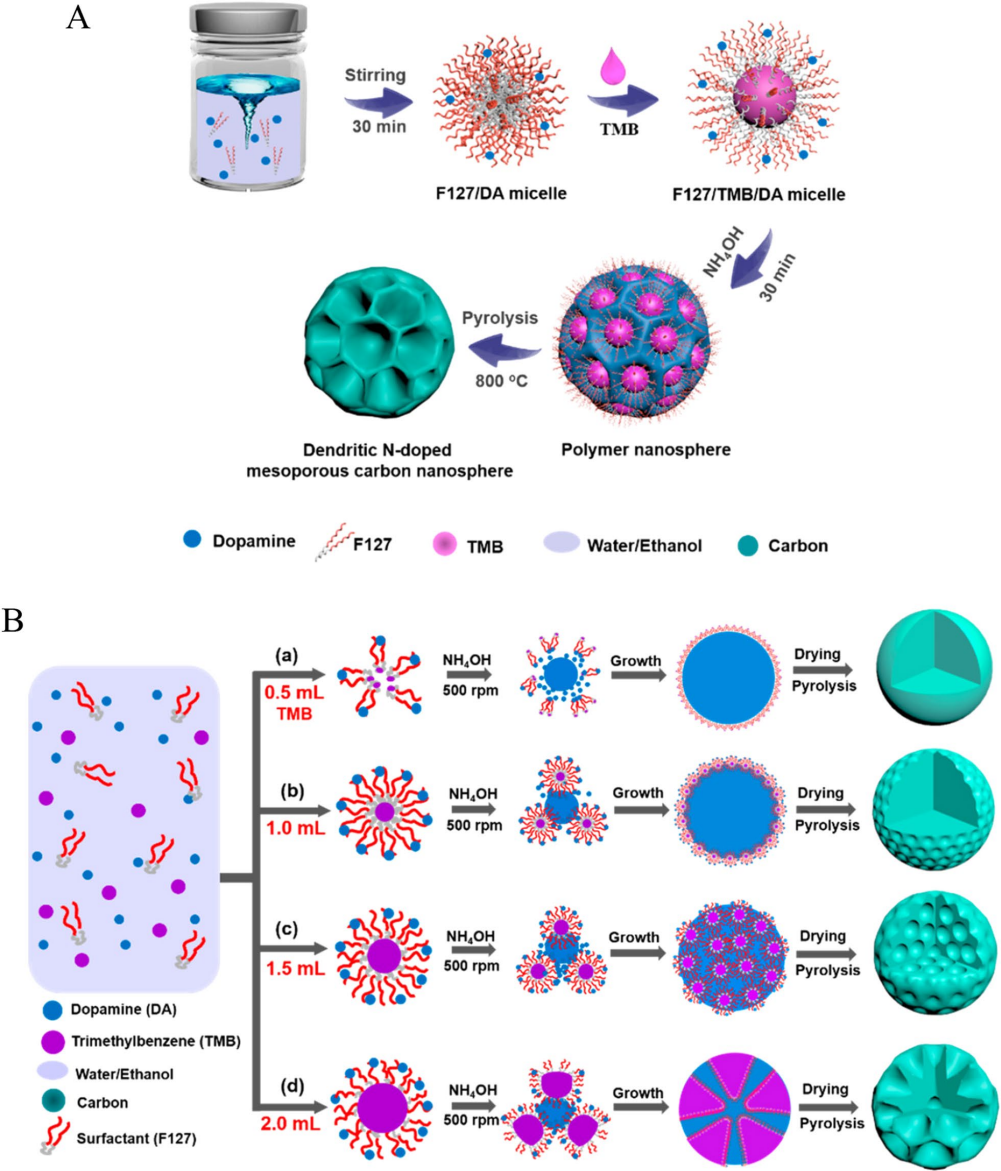
**A** Synthesis procedure for the dendritic N-doped mesoporous carbon nanospheres prepared by the versatile nanoemulsion assembly approach. **B** Schematic illustration of the formation process for the N-doped mesoporous carbon nanospheres with various morphologies and mesostructures prepared by the versatile nanoemulsion assembly approach. Reprinted (adapted) with permission from Peng et al. [65] (Copyright © 2019, American Chemical Society)

Soft templates generally refer to relatively soft surfactants, such as high-molecular-weight block copolymers or molecular aggregates, which have strong electrostatic and hydrogen bonding interactions with the precursors. The main characteristics of soft templates used for nanomaterial preparation include: (1) amphiphilic molecules, such as cetyltrimethylammonium bromide, polyoxyethylene-polybutene diblock copolymer, and triblock copolymers F127 or P123, which possess great advantages in simulating biomineralization; (2) diverse morphology; and (3) easy construction and simple equipment. Unlike the hard templating method, this method is based on synthesis at the molecular level. The chemical reaction between the template agent and the carbon precursor is an important factor in the synthesis process. Moreover, the ratio of reactants and temperature is important for porous carbon materials. Compared with hard templates, soft templates have a relatively simple synthesis process and cause less pollution to the environment. However, the preparation of porous carbon materials by the soft templating method also faces problems, such as low yield and low availability of template agents. Recently, the soft templating method has been widely used in the preparation of MPDA nanoplatforms [62–64]. Tang et al. [52] reported the synthesis of highly nitrogen-doped mesoporous carbon spheres (NMCS). By using polystyrene-block-poly (ethylene oxide) (PS-*b*-PEO) micelles as soft templates, they prepared PDA/PS-*b*-PEO composite spheres which was formed by self-polymerization of dopamine (DA) and spontaneous co-assembly with PS-*b*-PEO micelles. Finally, after carbonization and removal of the template, NMCS with high electrocatalytic activity and long-term stability were obtained, which was formed a carbon-based nanomaterial (ca. 200 nm) with a super-large mesoporous structure (ca. 16 nm). Peng et al. [65] adopted a versatile nanoemulsion assembly approach to prepare N-doped mesoporous carbon nanospheres with high uniformity and large tunable pore diameter. Using Pluronic F127 as the soft template, 1,3,5-trimethyl benzene (TMB) molecule as the modifier, and dopamine as the nitrogen and carbon source, a series of nitrogen-doped carbon nanospheres with adjustable pore diameter were synthesized in a water and ethanol system (Fig. 1). The authors found that in the synthesis process, small organic molecules (TMB) not only determined the size of the pores but also affected the interface formed by the soft template (F127) and the precursor (dopamine). Therefore, by adjusting the amount of TMB, the assembly method of micelles could be effectively controlled to adjust the pore size of the carbon balls. Smooth carbon balls, golf-shaped carbon balls, multi-cavity carbon balls, and dendritic carbon balls were obtained. The pore size of the carbon balls could be adjusted in the range of 5–37 nm. Dendritic carbon spheres had a relatively large pore size (~ 37 nm), small particle size (~ 128 nm), high specific surface area (~ 635 m^2^ g^−1^), and rich nitrogen content (~ 6.8 wt%). This simple and ingenious synthesis strategy of nano-microemulsion provided a new idea for the synthesis of novel nanostructures. Recently, Zhao et al. [66] developed a programmed shear-induced dynamic assembly method to prepare divergent gradient pore carbon nanospheres (MCSs) with a unique shell-core structure. The final synthesized MCSs had a divergent gradient pore structure, uniform particle size (~ 230 nm), high specific surface area (~686 m^2 ^g^−1^), large pore volume (~ 1.5 cm^3 ^g^−1^), rich N content (~ 6.7 wt%), and three-dimensional open structure. They used Pluronic F127 as the soft template, TMB molecule as the modulator, and dopamine as the nitrogen and carbon source. First, at a high stirring rate (500 rpm), TMB molecules entered F127/DA micelles to form large single micelles (F127/TMB/DA micelles). Under the action of alkaline catalyst (NH_4_OH), the large micelles assembled into the PDA core spheres with large mesopores. After the stirring rate was reduced to 300 rpm, small-sized F127/TMB/DA micelles appeared, continuously assembled on the PDA core, and finally fused together to afford PDA balls with a double mesoporous shell-core structure. By developing a programmed shear-induced dynamic assembly method, the authors intelligently synthesized MCSs with a unique core-shell and divergent gradient pore structure, opening a new path for the construction of highly complex and functional mesoporous materials. Lin et al. [67] also utilized the triblock copolymer Pluronic F127 and TMB as the soft template. The precise control of PDA nanosphere morphology was achieved by adjusting the weight ratio of TMB and F127. The prepared hollow MPDA nanospheres (H-MPDANS) exhibited good biocompatibility, excellent photothermal performance, high drug-loading capacity, and excellent sustainable drug release. Under NIR laser irradiation or acidic conditions, H-MPDANS enhanced drug release, allowing a combined treatment with chemotherapy and hyperthermia therapy. The nanospheres also showed good antitumor effect, revealing great potential for future clinical applications. In the soft template method, to obtain mesoporous materials with larger pore diameters, block copolymers with larger molecular weights are usually used as soft templates. Moreover, some macromolecular organics can be added to solubilize the soft templates to expand the pore size. Guan et al. [68] used the macromolecular block copolymers F127 and P123 as dual soft templates, TMB as the pore expander, and dopamine hydrochloride as the carbon source. MPDA nanoparticles having walnut-like, open, hierarchical pores (13 nm/50 nm) were synthesized in one step through nucleating growth on the dual soft template. Regarding the regulation of the pore structure, mesoporous materials with different pore structures were obtained mainly by controlling the assembly method of inorganic species and soft templates. In this study, the double soft template method was used to synthesize carbon nanospheres with special pore structure in one step. The synthesis strategy was simple and easily adjustable, providing a reference for the synthesis of other mesoporous materials. In addition, mesoporous C/N materials with open, multi-stage pores have certain advantages in the electrocatalytic reduction reaction of oxygen, presenting novel insights to the design and synthesis of catalysts for the electrocatalytic oxygen reduction reaction. Similarly, Xu et al. synthesized MPDA nanoparticles with good stability and suitable pore size by controlling the ratio of P123 and F127 [69]. Sialic acid-PEG-modified MPDA could specifically bind to E-selectin, which is followed by the release of indocyanine green and 3-(3-pyridyl)-1-(4-pyridynyl)-2-propen-1-one (3PO). Accordingly, PDT therapy was enhanced by inducing tumor blood vessel normalization. Utilizing its adhesion characteristics, PDA was wrapped on Fe^3+^, superparamagnetic iron oxide (SPIO), graphene oxide, and other materials [70–72]. Multifunctional MPDA composite nanomaterials were constructed, thus creating new prospects for the application of mesoporous dopamine in multiple fields.

## Mesoporous polydopamine applications in tumor therapy

**Fig. 2:**
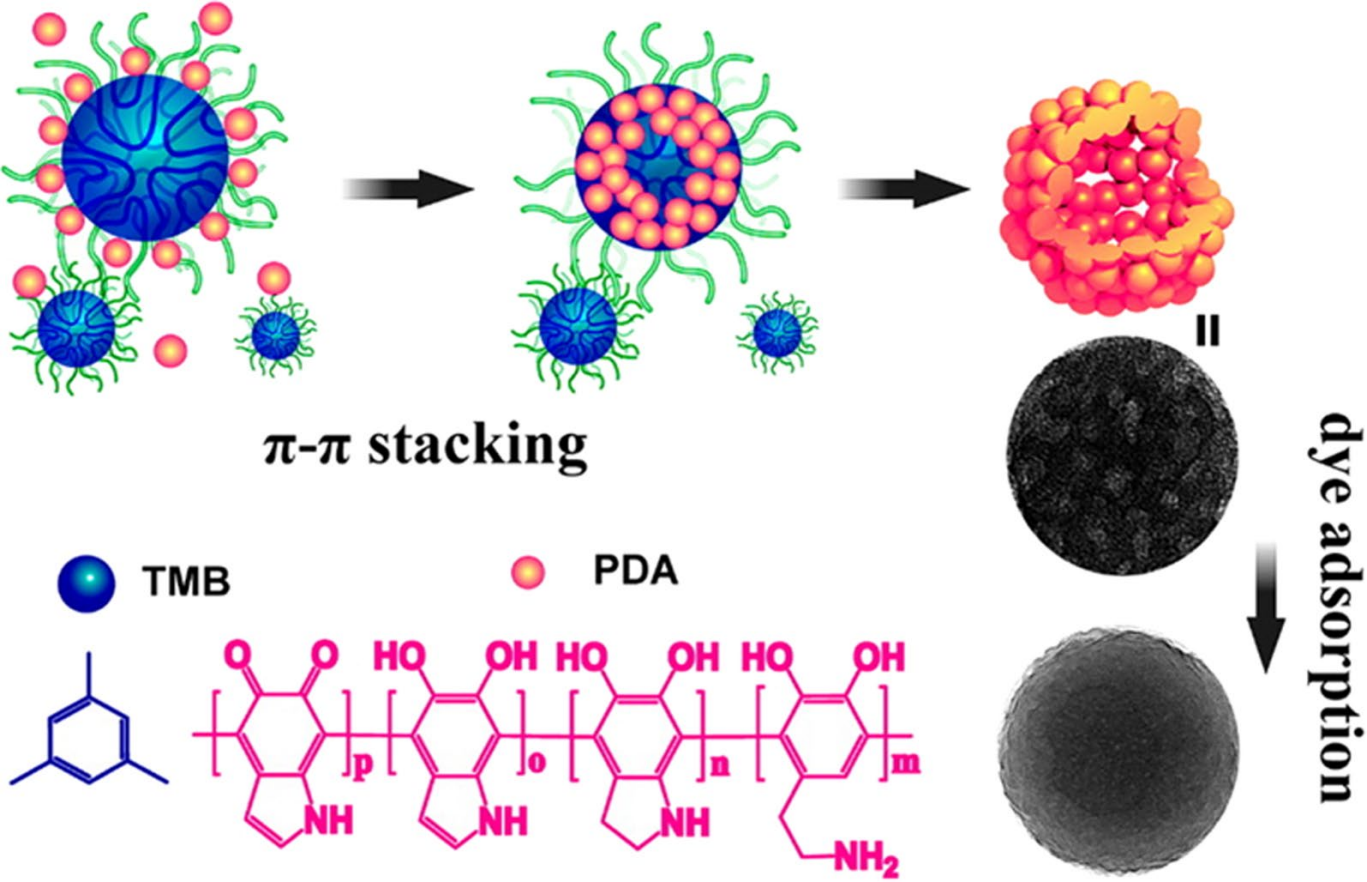
Preparation of mesoporous polydopamine and high adsorption performance. Reprinted (adapted) with permission from Chen et al. [76]. (Copyright © 2016, American Chemical Society)

Before the introduction of its mesoporous structure, PDA was mainly used for tumor photothermal therapy [73–75] owing to its effective photothermal conversion. However, conventional PDA nanoparticles have limited drug-loading efficiency. Moreover, the encapsulated drugs are easily dissociated under complex physiological conditions. In contrast, PDA nanoparticles with porous structures have significantly enhanced drug-loading efficiency and excellent curative effects. Chen et al. [76] reported a preparation method of MPDA nanoparticles using F127 and TMB as organic templates, ethanol as a cosolvent, and Tris as a catalyst for dopamine polymerization. The obtained MPDA had an average particle size of 90 nm, with slit-like pores (peak size, 5.0 nm). When used as an adsorbent model to adsorb rhodamine B (RhB), the MPDA particles achieved an ultra-high RhB adsorption capacity of 1100 µg mg^−1^, significantly higher than that of PDA-reactive dye with an Eschenmoser structure (Fig. 2). Accordingly, MPDA has shown great potential for drug loading. Xing et al. [77] synthesized MPDA nanoparticles using F127-stabilized emulsion droplets as templates. The average hydrodynamic size was 142 nm, and the peak pore size was 5 nm. Next, doxorubicin (DOX) and D-α-tocopherol polyethylene glycol 1000 succinate (TPGS, ATP-binding cassette transporter inhibitor) were absorbed on the nanoparticles via π–π stacking and hydrophobic-hydrophobic interactions. The prepared nanodrug delivery system exhibited high payloads of DOX (up to 2000 µg/mg). Benefiting from TPGS-mediated multidrug resistance reversal, the delivered DOX exerted strong antitumor effect on MCF-7/ADR cells. Moreover, MPDA converted NIR light into fatal heat, thereby killing the cancer cells.

Wang et al. [78] prepared MPDA-calcium phosphate composites by loading calcium phosphate on the surface. The surface of the particles was modified with tertiary amines via the facile Michael addition/Schiff base reaction of PDA to afford high siRNA-loading capacity (10 wt%). siRNA cytoplasmic delivery and release, as well as synergistic treatment with photothermal conversion-gene drug, were thus completed. As a mesoporous organic polymer, MPDA possesses not only the mesoporous silicon structural characteristics but also the diversified modifiable and functional properties of the polymer. Additionally, PDA has excellent optical and electrical properties. Therefore, MPDA offers promising applications and research potentials in cancer diagnostics and nanomedicine.

### Application of mesoporous polydopamine in chemo-radiotherapy

**Fig. 3:**
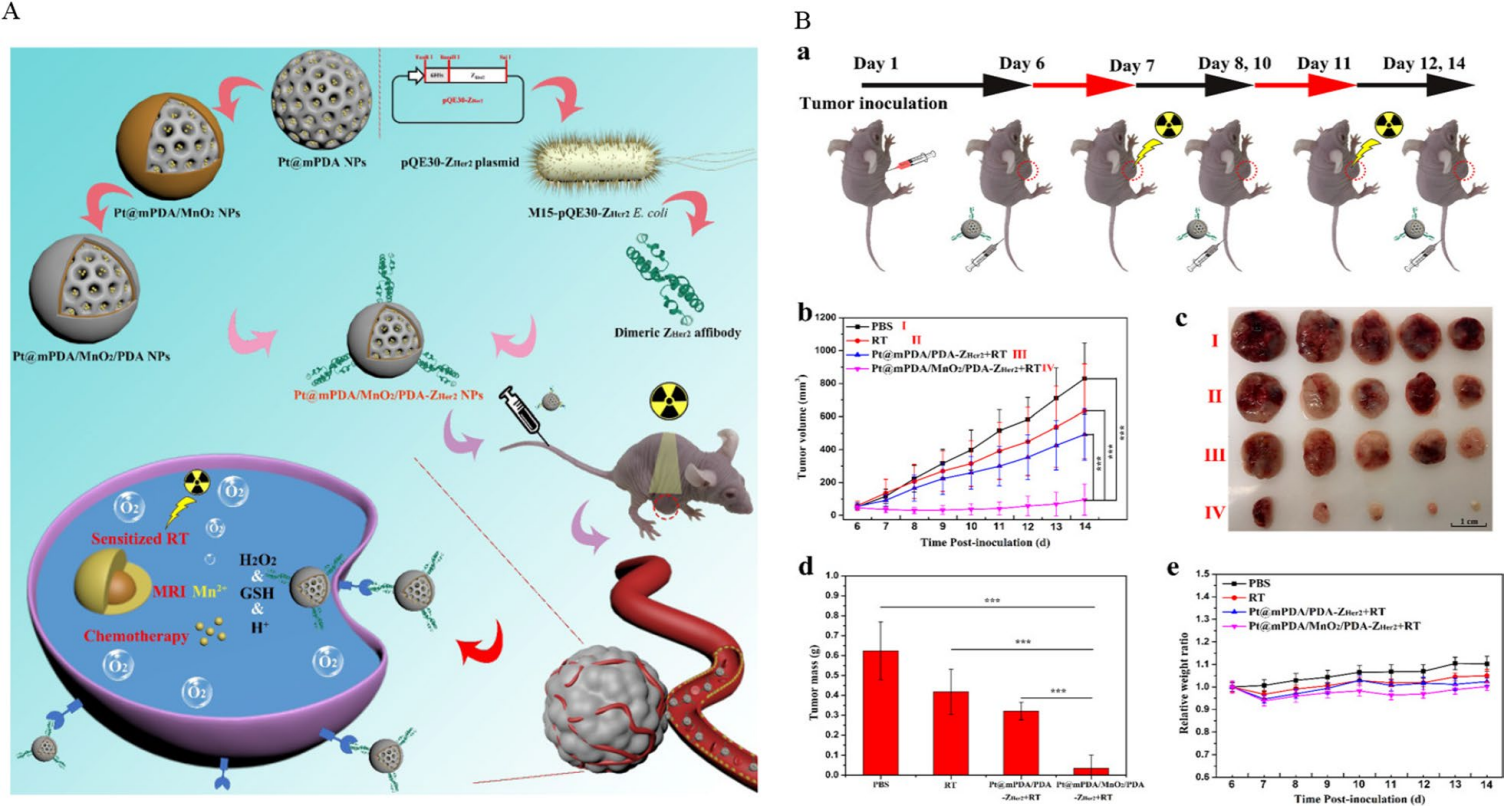
**A** Preparation of Pt@mPDA/MnO_2_/PDA-Z_Her2_ NPs and in vivo MRI-guided enhanced chemo-radiotherapy. **B** **a** Schematic illustration of process of the chemo-radiation combined therapy; **b** SKOV-3 tumor growth curves of different groups after intravenously injection of the formulations; **c** The images of tumors isolated after treatment for 8 days; **d** Mean weights of the tumors isolated on day 14; **e** Body weight changes over the 8 days of the experiments. Reprinted (adapted) with permission from Wang et al. [83]. (Copyright © 2021 Wang et al. Journal of Nanobiotechnology published by BioMed Central). http://creativecommons.org/licenses/by/4.0/

Radiotherapy is a widely used treatment that adopts high-energy rays to inhibit the proliferation of cancer cells. However, the high-energy rays inevitably induce damage to the normal tissues, exerting hazardous effects related to radiotherapy [79]. In addition, oxygen plays an important role in the process of ionizing radiation and killing of tumor cells, but the tumor microenvironment (TME) in solid tumors is threatened by oxygen depletion. All these features negatively affect the therapeutic efficacy of radiotherapy in clinical setting [80–82]. Considering the shortcomings of this single treatment, combination therapy may lead to improved therapeutic effect. Radiotherapy combined with chemotherapy represents a new strategy to overcome the above disadvantages. Wang et al. [83] integrated Her2 targeting ability as well as diagnosis and radiotherapy sensitization into one platform, offering a novel approach for translational tumor theranostics. The constructed mPDA NPs had an average size of 119 nm and a pore size of 2 nm. Next, cisplatin was loaded by electrostatic force between the negatively charged mPDA and the positively charged cisplatin, affording Pt@mPDA NPs. Utilizing the reducibility of PDA, the oxidation-reduction reaction between PDA and KMnO_4_ produced manganese dioxide (MnO_2_). MnO_2_ was coated on the outer layer of Pt@mPDA NPs and used to decompose endogenous H_2_O_2_ to produce O_2_, thereby alleviating tumor hypoxia and increasing tumor sensitivity to radiotherapy. To improve the biocompatibility and targeting of Pt@mPDA/MnO_2_, a biomimetic PDA layer and Z_Her2_ affibodies were introduced. Finally, a smart Pt@mPDA/MnO_2_/PDA-Z_Her2_NP was constructed. The results showed that Pt content in the Pt@mPDA/MnO_2_/PDA-Z_Her2_NP that accumulated in mouse tumors was 3.5 times higher than free Pt content. Furthermore, immunofluorescence analysis showed that a decrease in hypoxia-inducible factor 1α (HIF-1α) expression promoted sensitivity to radiotherapy. This multifunctional and intelligent nano-platform provided a theoretical basis for clinical transformation (Fig. 3).

### Combination therapy associated with photothermal therapy

#### Photothermal therapy combined with chemotherapy

Unlike traditional treatments, which can cause various side effects and drug resistance, photothermal therapy act as an alternative and supplementary treatment for conventional cancer treatment owing to its low invasiveness, fewer complications, spatiotemporal light control property, and fast recovery period [84–86]. An ideal photothermal agent is capable of strong absorption in the NIR region, with low toxicity and good biocompatibility. When irradiated by an NIR laser, it can convert the absorbed light energy into heat energy and thus cause the local temperature to rise, thereby destroying the cell membrane and denaturing the protein around cancer cells. The structure of PDA is similar to that of natural eumelanin. Owing to its strong absorption in the NIR spectral range, PDA is commonly used as a photothermal reagent, with high photothermal conversion efficiency (40%) and good curative effect [87]. However, long-term application of a single photothermal therapy may cause the overexpression of heat shock protein, leading to the heat tolerance of tumors. Combination therapy, such as chemo-photothermal therapy, is widely used to overcome this obstacle [88–90]. Zhang et al. [91] synthesized PEG (polyethylene glycol)-modified MPDA (MPDA-PEG) via an emulsion-induced interface assembly method. The prepared MPDA-PEG had high photothermal conversion efficiency (~ 41%) and high paclitaxel (PTX)-loading content (PTX, DLC = 15%) (DLC, drug-loading content). As the temperature increased, PTX release was accelerated. Compared with chemotherapy alone and photothermal therapy (PTT) treatment alone, PTX combined with PTT (laser irradiation at 1 Wcm^−2^ for 10 min) resulted in an antitumor rate of up to 93%. Following MPDA-PEG-PTX treatment combined with 2 Wcm^−2^ laser irradiation for 5 min, the tumor showed almost complete ablation, and no obvious systemic toxicity caused by the nanoformulation was observed during the treatment. Therefore, this simple photothermal nanomaterial technology with good biocompatibility and biodegradability has significant therapeutic value for tumor treatment. In tumor tissues, as tumor cells produce a large amount of lactic acid via glycolysis, the TME becomes weakly acidic, with a pH range of 6.0–7.0 [92, 93]. This characteristic pH is often used to selectively trigger some nanocarriers to enhance the effectiveness of cancer treatment. PDA has good pH responsiveness. A PDA-modified membrane remains stable in the neutral pH environment, but is degraded in the acidic environment of tumors, thus releasing the loaded drug [21, 22]. Chen et al. [94] constructed self-coated nanoparticles formed by MPDA and PDA, and modified it with hyaluronic acid (HA). They loaded the antitumor drug docetaxel (DTX) to construct the MPDA-DTX@PDA-HA (MD@PH) nano-targeting system. The MD@PH nano-targeting system had high photothermal conversion efficiency (26.6%) and pH-sensitive drug release characteristics. The in vivo results showed that the MD@PH + NIR group showed the lowest tumor volume, with the tumor almost completely ablated, which proved the effectiveness of combined treatment with PTT and chemotherapy. Both the core and the shell of MD@PH were composed of PDA, which simplified the preparation method, prevented potential biocompatibility problems, and greatly increased the possibility of its development from laboratory research to clinical application.

**Fig. 4:**
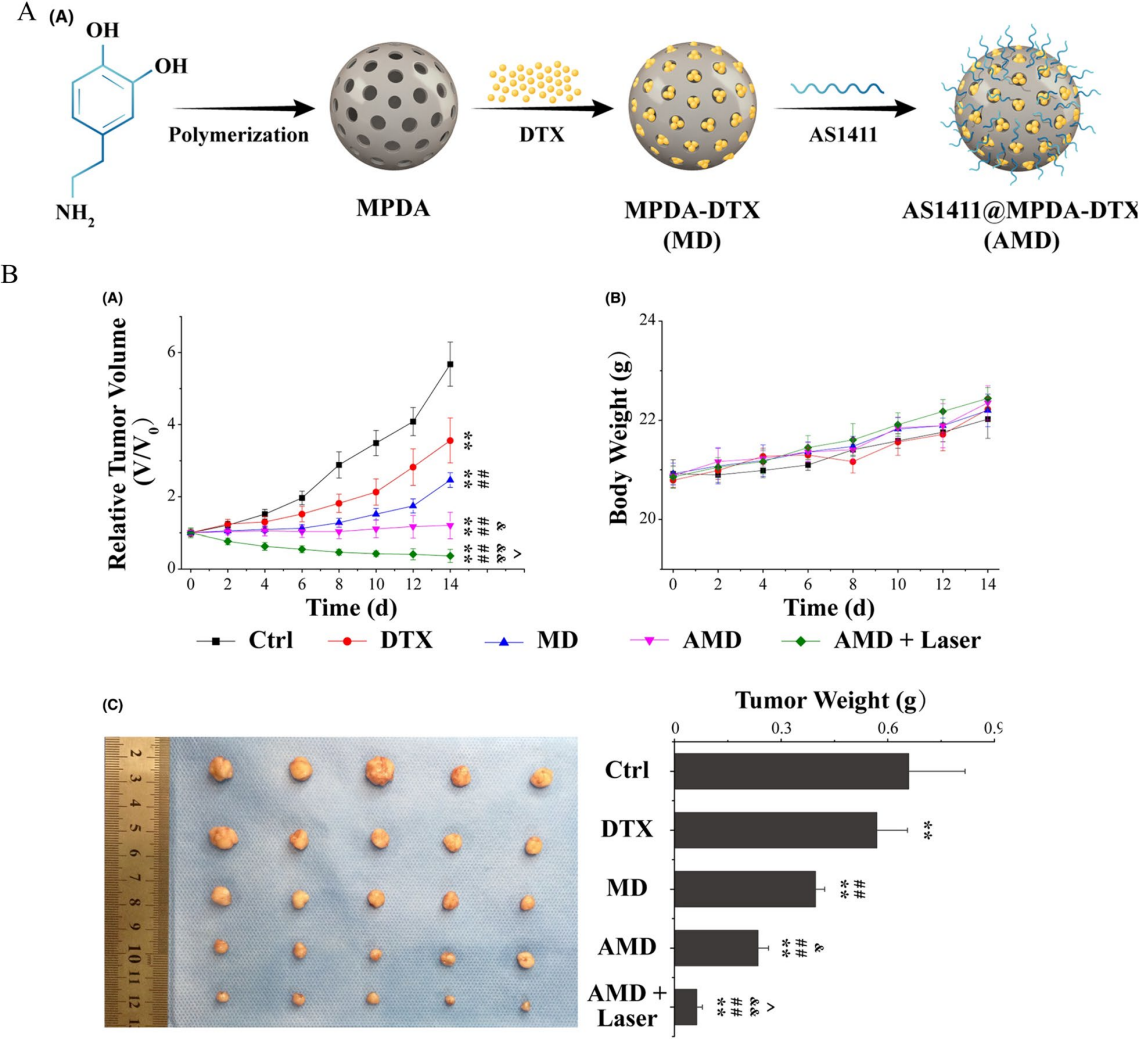
**A** Schematic illustration of synthesis of AMD. **B** **a** Relative tumor volume of PC-3 tumor-bearing mice with different treatments. **b** Body weight of tumor-bearing mice with different treatments. **c** Photographs and weight of tumors dissected from each group on the 14th day after different treatments. Reprinted (adapted) with permission from Dai et al. [99]. (Copyright © 2021 Dai et al. Cell Proliferation published by John Wiley & Sons Ltd)

The main concern in the delivery of targeted nano-drugs is the specific recognition and combination between the targeting ligand and the receptor, such as peptides, cytokines, aptamers, and antibodies. An aptamer has a high affinity for a target molecule and exerts an antagonistic effect on the specific function of the target molecule, and it can be used as a probe, drug, and drug carrier. Aptamer-modified nanomedicines are also widely used in cancer treatment [95, 96]. Owing to its rich chemical groups, MPDA can be functionalized through many functional molecules, such as targeted HA, amphipathic polyethylene glycol, and nucleophilic thiols [97, 98]. Dai et al. [99] synthesized MPDA nanoparticles via a one-pot method. By modifying AS1411 aptamers, they constructed a MPDA multifunctional nano-platform for targeted delivery of DTX(docetaxel) and for chemo-photothermal therapy against prostate cancer (AS1411@MPDA-DTX [AMD]) (Fig. 4). In vitro experiments revealed that incubation with MPDA-DTX (MD) nano preparations increased the proportion of apoptotic cells by approximately 16%. In contrast, the apoptotic rate after AMD treatment was approximately 25%, and AMD combined with laser treatment induced approximately 53% cell deaths, indicating that synergistic combination therapy with AMD and laser treatment significantly inhibited cancer cells. Moreover, in vivo results showed that combined treatment with AMD-mediated chemotherapy and PTT suppressed prostate cancer growth. Such a nanosystem may lead toward the development of synergistic chemo-photothermal therapy against prostate cancer.

#### Photothermal therapy combined with immunotherapy

Tumor immunotherapy has become the most powerful cancer therapeutic approach accessible [100]. However, it is greatly limited by immune evasion and the lack of tumor tissue microenvironment to stimulate immune response signals. Recently, a combination of photothermal and immunotherapy based on nanoparticles has exhibited strong efficacy. PDA, benefiting from inherent strong NIR absorption, has been reported to possess high-efficiency photothermal conversion ability to directly destroy tumor cells, and release tumor antigens to enhance tumor immunogenicity. Huang et al. [101] designed a nano-platform with mesoporous silica nanoparticles (MSNs) as a carrier. This strategy integrated the photothermal reagent PDA, model antigen ovalbumin (OVA), and antigen promoter ammonium bicarbonate (ABC), to successfully construct the MSNs-ABC@PDA-OVA nano-vaccine for combined photothermal/immune treatment of melanoma. The MSNs-ABC@PDA-OVA nano-vaccine showed excellent photothermal properties and effectively ablated the primary tumor. Under laser irradiation, the MSNs-ABC@PDA-OVA nano-vaccine also achieved rapid antigen release and endosomal escape, enhanced the activation and maturation of dendritic cells, and promoted their migration to tumor-draining lymph nodes, thereby inducing powerful antitumor immune response. Experimental results showed that a single injection of MSNs-ABC@PDA-OVA combined with single photothermal treatment successfully eradicated melanoma, with a cure rate of 75%, and generated strong immune memory to inhibit tumor recurrence and lung metastasis. Thus, this research provides a simple and promising new collaborative strategy for cancer treatment. With the development and wide application of PDA in the field of biomedicine, MPDA has also received great attention. Wang et al. [102] studied MPDA nanoparticles (MPDA NPs) for lymph node-targeted immune activation. With the TLR7 agonist imiquimod (R837) as the immune regulation model, an MPDA-based drug-delivery system was designed and applied for targeted lymphatic immune activation. The MPDA surface was modified by the biocompatible polymer polyvinylpyrrolidone (PVP) to enhance its lymphatic drainage ability. Compared with free R837, this nano-adjuvant possessed obvious advantages in promoting the maturation of dendritic cells while being transported to and retained in the proximal lymph nodes, which greatly increased drug exposure in the lymphatic sites. Effective dendritic cell activation and CD8^+^ T cell response were observed when R837 was released in the lymph nodes. Endogenous antigen was released from tumor apoptosis induced by the photothermal effect of MPDA, which further enhanced this effect, resulting in the inhibition of B16 melanoma. In the B16 melanoma model, thermal ablation of tumor cells via apoptosis and production of cytotoxic T lymphocytes effectively inhibited tumor growth. Overall, the combination of photothermal therapy and immunotherapy based on the PVP-MPDA@R837 nano-platform had great potential in the treatment of melanoma.

#### Photothermal therapy combined with photodynamic therapy/chemodynamic therapy

Photodynamic therapy (PDT) has gained great attention as a new method of cancer treatment owing to its effectiveness, safety, synergy, reproducibility, and relatively low treatment cost [103, 104]. PDT comprises of a photosensitizer, a light source of specific wavelength, and sufficient oxygen. The photosensitizer can produce reactive oxygen species such as singlet oxygen, which is cytotoxic, and release a series of cytokines to destroy tumor cell membranes and capillaries under specific wavelength light irradiation, thereby killing tumor cells. Because both PDT and PTT can be excited by NIR light, they are often used in combination [105, 106]. In this combined usage, PDT needs to consume oxygen, but the TME is often hypoxic; thus, the problem of oxygen delivery in PDT must be solved. Hu et al. [107] established a multifunctional MPDA-based nano-platform. Platinum nanoparticles (Pt NPs) were modified in situ on the MPDA surface and used as catalysts to decompose endogenous H_2_O_2_ to produce O_2_. This nanosystem could overcome tumor hypoxia and enhance PDT efficacy. The nano-preparation was modified by bovine serum albumin (BSA) to improve the dispersion and stability. With DOX as an antitumor drug and Chlorin e6 (Ce6) as a photosensitizer, and by integrating catalase-like Pt nanozymes and MPDA, M-Pt-BCD nano-preparation was obtained. This nano-preparation showed a high loading capacity for DOX (18.2 %) and Ce6 (36.1 %), as well as high light-to-heat conversion efficiency (~ 23%). In vivo experiments revealed that M-Pt-BCD nanoformulations showed significant antitumor efficacy in combination with DOX/PDT (650 nm)/PTT (808 nm). The tumor volume was reduced by nearly 50 %, and the preparation had almost no obvious systemic toxicity. This MPDA-based nano-platform provides ideas for the development of technologies for combination cancer treatments.

Different from the conventional decomposition of oxygen in the TME and exogenous delivery of oxygen, Xu et al. [69] constructed sialic acid (SA)-modified PEG-capped MPDA nanoparticles (SA-PEG-MPDA). The photosensitizer indocyanine green and the PFKB3 kinase inhibitor 3PO were co-loaded on the SA-PEG-MPDA nanoparticles to obtain a multifunctional MPDA nanodrug delivery system. The nanodrug delivery system, which could efficiently target the tumor site, was then irradiated by NIR light, which can effectively convert light energy into singlet oxygen and heat energy. This combined therapy with PDT and PTT effectively killed tumor cells and gradually released 3PO to repair the abnormal tumor vascular system, normalizing the TME and improving the oxygen supply capacity of the tumor site, thereby fundamentally solving the problem of insufficient oxygen due to hypoxia in the later stage of PDT and achieving long-term sustainable antitumor effects.

**Fig. 5:**
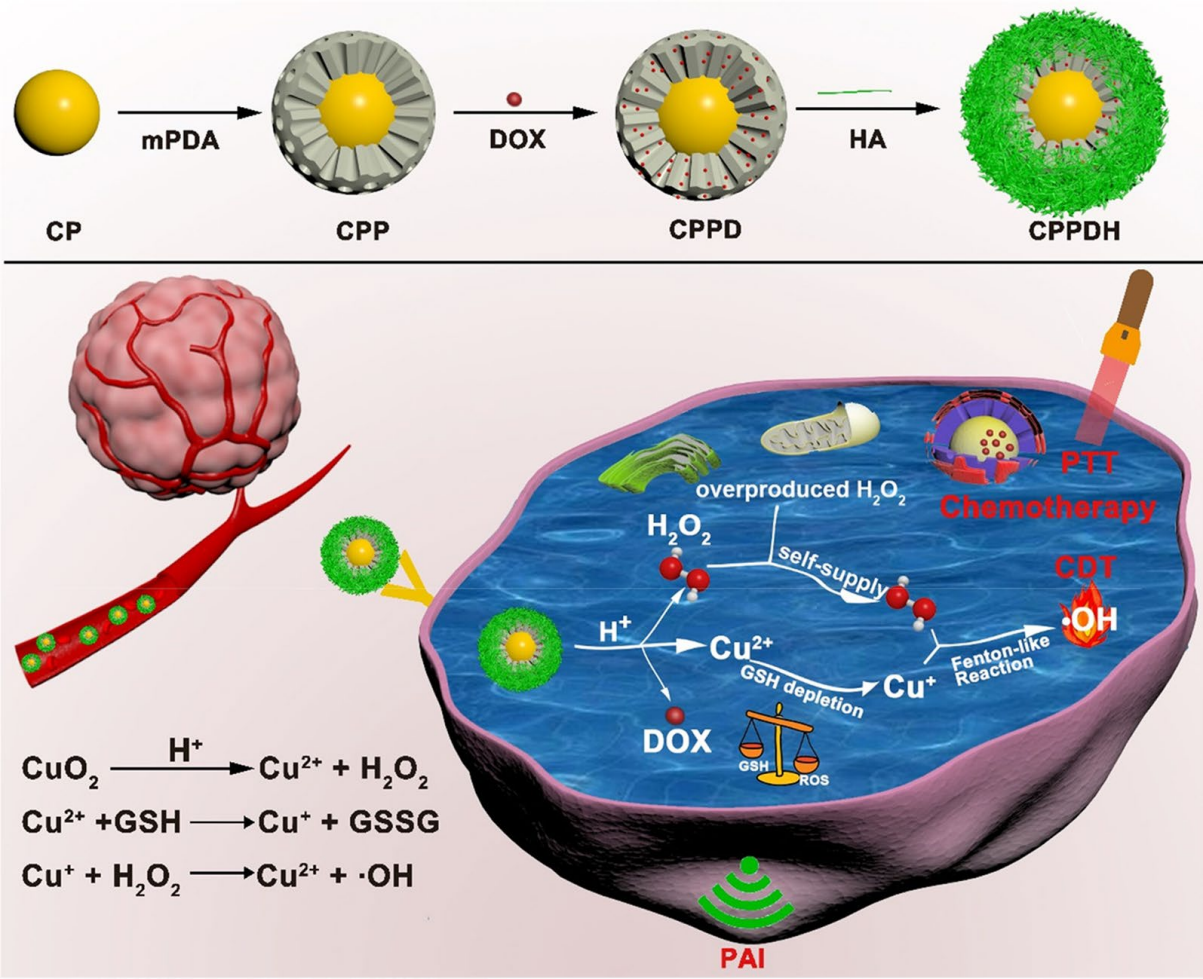
Synthetic Sequence of CPPDH and Schematic Diagram of the Therapeutic Mechanism of CPPDH for Synergistic Self-Enhanced CDT, Photothermal, and Chemotherapy. Reprinted (adapted) with permission from Xiao et al. [110]. (Copyright © 2021, American Chemical Society)

Owing to the limited penetration depth of light, PTT alone is difficult to completely eliminate cancer cells and prevent tumor recurrence. Considering that cancer cells are more susceptible to oxidative damage than normal cells, stimuli are introduced to activate oxidative stress to disrupt the redox balance, ultimately resulting in therapeutic effects. Chemodynamic therapy (CDT) is a treatment method that utilizes hydroxyl free radicals (·OH) produced by tumor in situ Fenton reaction or Fenton-like reaction to kill tumor cells. It is only activated by endogenous substances and has the advantages of high regional selectivity, which is an emerging cancer treatment to overcome the limited penetration depth of light [108, 109]. As temperature has a certain effect on the rate of chemical reactions, these chemical reactions also include Fenton/ Fenton-like reactions. In view of this, when PTT is combined with CDT, a high temperature will accelerate ·OH production, thereby improving the therapeutic effect. Xiao et al. [110] constructed MPDA on the non-spherical surface of copper peroxide (CuO_2_, CP) via a seed-mediated microemulsion method, using TMB and the triblock copolymer Pluronic F127 as pore swelling agents and pore templates, respectively, yielding CP@mPDA (CPP). CPP was modified with HA through electrostatic adsorption, and the anticancer drug DOX was loaded by π-π stacking to obtain CuO_2_@mPDA/DOX-HA (CPPDH) multifunctional nano-platform integrating CDT, PTT, and chemotherapy, which could induce the depletion of glutathione (GSH) and self-supply of H_2_O_2_. CuO_2_ nanodots were specifically triggered by the acidic TME to produce a large amount of Cu^2+^ and H_2_O_2_. When HA specifically recognized the CD44 highly expressed on the tumor surface, Cu^2+^ release and H_2_O_2_ production were triggered by the acidic environment of lysosomes. Subsequently, the Cu^2+^ was reduced by GSH into Cu^+^, which in turn catalyzed H_2_O_2_ to produce ·OH. The generation of ·OH was distinctly enhanced by GSH depletion and H_2_O_2_ self-sufficiency. In vitro studies revealed that cells treated with only laser irradiation or free DOX showed only slight damage, whereas some cells died after CP treatment, and almost all cells died after CPPDH+laser treatment (laser: 808 nm 1 W/cm^2^ for 2 min). In vivo experiments also revealed that CPPDH+laser treatment (laser: 808 nm 1 W/cm^2^ for 5 min) resulted in significant tumor suppression and caused no significant systemic toxicity. The research proposes self-boosting CDT combined with photothermal and chemotherapy as a new strategy for cancer therapy (Fig. 5).

In addition, the high photothermal conversion performance of MPDA was applied in combination with autophagy blocking for improved antitumor efficacy. Huang et al. [111] adopted autophagy blocking to enhance the sensitivity of cells to photothermal therapy. During photothermal therapy, cells underwent autophagy to resist heat damage. However, NIR triggered the release of the autophagy inhibitor chloroquine from the nanosystem, which inhibited PTT-induced protective autophagy of cancer cells, thereby increasing the sensitivity of tumor cells to heat and improving the antitumor efficacy of photothermal treatment.

## Cancer theranostics

Cancer theranostics has always been a problem worldwide, and traditional chemotherapy and radiotherapy have significant side effects. Therefore, there is an urgent need to develop new safe and effective cancer theranostics methods. Along with technological advances in material science and chemistry, a broad range of theranostic nanoplatforms has been designed for imaging-guided cancer therapy. Nanotechnology is an effective method for delivering contrast agents into the targeted tissue, which can improve diagnostic imaging accuracy while causing fewer adverse effects. PDA, as a melanin-like polymer, has attracted attention in the field of biomedicine owing to its inherent biocompatibility, easy preparation, and strong NIR absorption characteristics. On account of the high specific surface area, regular and orderly pore structure, and excellent characteristics it retained from PDA, MPDA has also gained the interest of researchers [112, 113]. Magnetic resonance imaging (MRI) has become an important method of clinical disease diagnosis because of its high tissue-resolution, multi-parameter, and multi-directional imaging. Gadolinium is a common commercial MRI contrast agent with relatively good reflexivity and low biotoxicity. It has been widely used in the field of biomedicine for cancer theranostics [114–116]. However, owing to its own shortcomings, its application can occasionally cause patient to develop nephrogenic systemic fibrosis. To overcome this limitation, manganese is a good substitute because of its inherent advantages, such as low intrinsic toxicity and effective positive contrast enhancement effect [117, 118]. Wu et al. [119] synthesized MPDA NPs (called MPDAPs) using the soft template method and then complexed Mn^2+^ to obtain MPDAPs/Mn. In vitro infrared imaging analysis revealed that the photothermal conversion efficiency of MPDAPs/Mn was as high as 45.2%. Thermogravimetric analysis results showed that the Mn content in MPDAPs/Mn was 3.16%, which was approximately 2.5 times higher than that in PDAPs/Mn. Brunauer-Emmett-Teller and Barrett-Joyner-Halenda test results showed that the Brunauer-Emmett-Teller surface area of ​​MPDAPs/Mn was 45.7 m^2^ g^−1^, which was nearly 10 times higher than that of PDAPs/Mn (4.4 m^2^ g^−1^). The pore size of MPDAPs/Mn was mainly distributed at 3.4 nm, whereas that of PDAPs/Mn exhibited nonporous characteristics. These data showed that MPDAPs/Mn particles had higher specific surface areas than PDAPs/Mn. According to MRI data, the r_1_ of MPDAPs/Mn solution was calculated to be 10.08 mM^−1^ S^−1^, which was nearly 2 times higher than that of the commercially available MRI contrast agent Gd-DTPA (4.50 mM^−1^ S^−1^, 0.5 T), thus meeting the requirement for clinical applications. After injection of MPDAPs/Mn through the tail vein, the signal intensity at the tumor site gradually increased and reached the peak at 8 h, which was approximately 2.1-fold higher than that before injection. In comparison, the signal intensity of PDAPs/Mn in the tumor site was only 1.5 times higher than that before injection. The in vitro antitumor assay results also showed tumor suppression in the MPDAPs/Mn+NIR group, with the tumor weight 0.4 times lower than that in the PDAPs/Mn+NIR group, and MPDAPs/Mn exerted low toxicity to normal cells and organs. This study showed that the nano-platform combined with MRI-guided photothermal therapy has a good prospect in antitumor treatment.

**Fig. 6:**
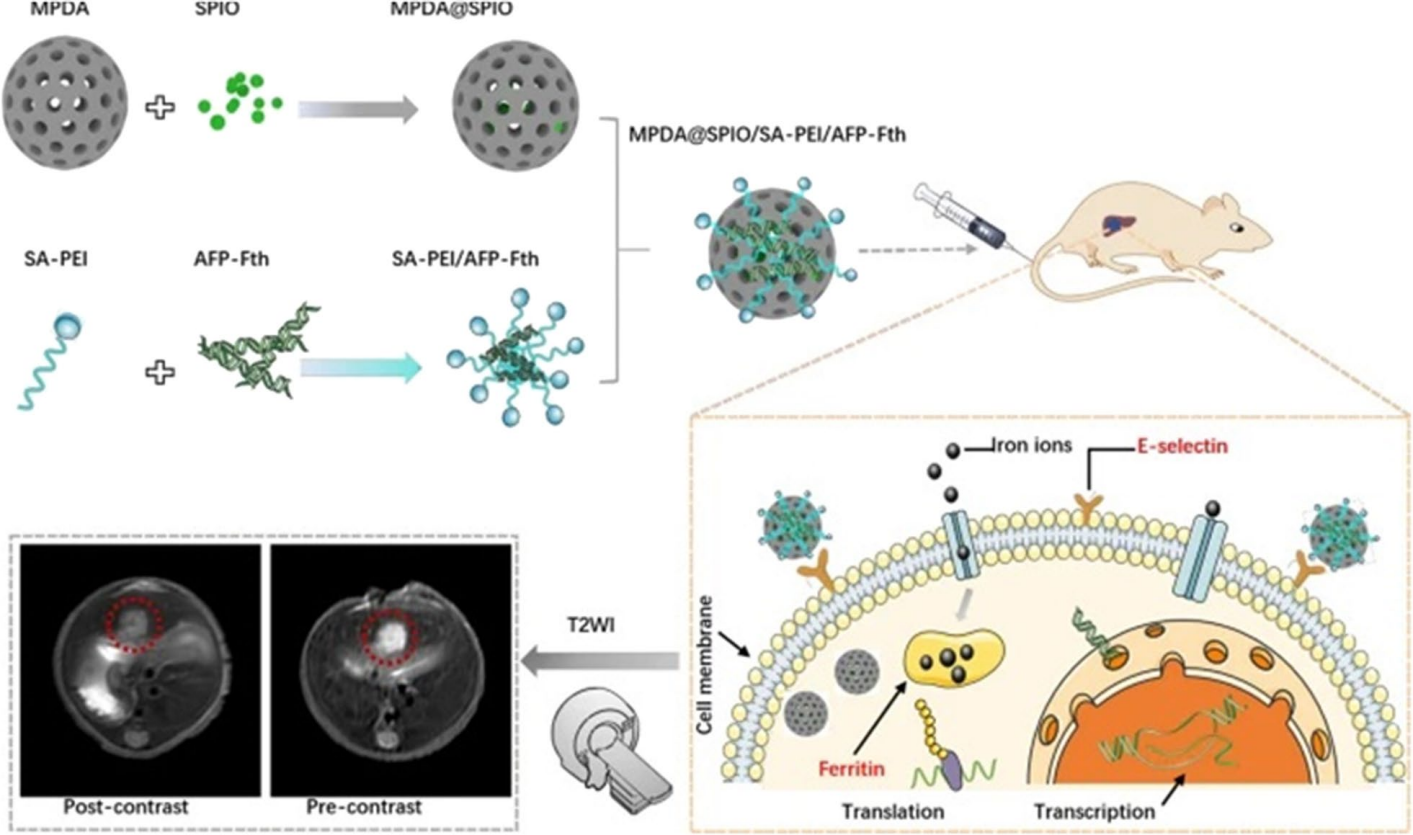
The application of MPDA@SPIO/SA-PEI/AFP-Fth nanocomplexes as an effective MR contrast agent in the early stage of HCC. Reprinted (adapted) with permission from Fan et al. [121]. (Copyright © 2021Fan et al. Journal of Nanobiotechnology published by BioMed Central). http://creativecommons.org/licenses/by/4.0/

To overcome the limitation of Gd application in patients with renal insufficiency, SPIO has emerged because it can be used in patients with renal impairment. It functions in cell metabolism and protein synthesis, such as the regular iron metabolism route in the body. Guan et al. [120] have made important progress in the study of MRI-guided tumor iron death and photothermal combination therapy. Ferroptosis is a non-apoptotic form of regulated cell death, in which the cell death mechanism was to generate and accumulate reactive oxygen species through the iron-based Fenton reaction to mediate cell death. The construction of nano drug delivery system possessing high drug-loading capacity and stimulus responsiveness characteristics and can be combined with imaging guidance technology is crucial for cancer theranostics. In this study, sorafenib (SRF) and ultrasmall SPIO nanoparticles were loaded to MPDA NPS to form a TME (GSH, H^+^, H_2_O_2_)-responsive multifunctional SRF@MPDA-SPIO nano-diagnostic/therapeutic agent. SRF, which is one of the few clinically approved drugs reported to induce iron death, was loaded on the pores and surface of MPDA via π-π accumulation and hydrophobic force. SPIO was loaded on the inside and surface of the MPDA channel via hydrophobic force. The iron in SPIO nanoparticles was released in the form of ferrous (Fe^2+^) or iron (Fe^3+^) ions in acid lysosomes, and then participated in the Fenton reaction, thereby inducing iron death of tumor cells. In addition, the MPDA carrier, as an efficient photothermal conversion agent (η = 38.1 %), allowed photothermal therapy. Through MRI guidance, the accumulation of the diagnostic/therapeutic agent in the tumor was dynamically monitored, achieving a high-efficiency combination cancer treatment with iron death and photothermal therapy. On this basis, Fan et al. [121] introduced the endogenous contrast agent ferritin, which included ferritin heavy chain (Fth) and light chain. Fth, the major regulator of ferritin activity, displayed ferroxidase activity to promote iron oxidation and chelation, and thus can be used as a T_2_-weighted image (T_2_WI) endogenous contrast agent. In this study, to prepare a dual-target T_2_-weighted MRI contrast agent MPDA nanocomposite (MPDA@SPIO/SA-PEI/AFP-Fth), MPDA was used as a carrier, SPIO as an exogenous contrast agent, alpha-fetoprotein regulated ferritin gene (AFP-Fth) as an endogenous contrast agent, and SA as a targeting moiety (Fig. 6). The prepared nanocomposite had good biocompatibility to liver cells. Following tail vein injection of the nano-preparation (the parameters of transverse T_2_-weighted fast spin sequence were TR, 3000 ms; TE, 73.5 ms; FOV, 160 × 100 mm; Matrix, 200 × 176; slice thickness, 1.5 mm), from 6 to 12 h, the signal-to-noise ratio (SNR) of the mice treated with MPDA@SPIO/SA-PEI/AFP-Fth nanocomposite decreased significantly from 48.8 to 32.3. In contrast, while there was much less change in the group of MPDA@SPIO/PEI/AFP-Fth (48.9–46.6). At 12–24 h, the SNR of tumors treated with MPDA@SPIO/SA-PEI/AFP-Fth nano preparations was still weakening, and the tumor slices were in 24 h showed SNR=25.2, when ferritin expression reached the level required for measurable T_2_W1 effect, Fth reporter gene with AFP promoter could be worked. In 24–48 h, the tumor SNR of MPDA@SPIO/SA-PEI/AFP-Fth was maintained at 25.3–28.3. The results showed that compared with other preparations, MPDA@SPIO/SA-PEI/AFP-Fth had better ability to enhance MRI contrast of T_2_-weighted images in tumor region, and could be used as an effective MRI contrast agent for early detection of hepatocellular carcinoma (HCC).

To change the signal intensity of the tumor site and improve the accuracy of MRI theranostic, MRI contrast agents are essential. According to the mechanism, contrast agents are mainly categorized into T_1_ and T_2_. The application of T_1_/T_2_ dual-mode MRI contrast agent can overcome the deficiency of a single contrast agent in accurate tumor detection. It can accomplish various imaging results for dual cancer theranostics, and it will play a critical role in the accurate qualitative and precise positioning of cancer. Shu et al. [71] developed a novel MPDA-based theranostic agent for T_1_/T_2_ dual MRI-guided cancer chemo-photothermal therapy. They prepared MPDA NPs loaded with SPIO, and used SA as a targeting ligand, MPDA-chelated Fe^3+^, and DOX as the antitumor drug. This nano-platform was highly effective for cancer theranostics owing to the precise guidance of dual-mode MRI.

Ultrasound (US) imaging is a commonly clinical diagnosis method. It has the advantages of real-time monitoring, high safety, high sensitivity, low cost, and portability. However, its resolution is relatively low, and it is easily interfered by gas. Computed tomography (CT) imaging has a good spatial resolution to overcome the shortcomings of US imaging, and it is not interfered by gas and bone. Moreover, CT imaging has better tissue penetration, is more cost-effective, and can perform three-dimensional vascular reconstruction. Thus, US/CT dual-modal imaging is highly promising for improving real-time imaging contrast, acquiring clinical application anatomical information, and guiding and monitoring tumor ablation [122–124]. Yuan et al. [125] developed a multifunctional nanodrug delivery system with multiple therapeutic and imaging functions. Aminoborane was loaded onto MPDA NPs as a prodrug for hydrogen production, and PEG was modified on the surface of the nanoparticles. The H_2_ gas generated by aminoborane in the weak acid conditions of the TME not only was used to treat tumors via combined hydrogen and photothermal therapy but also serves as a US/CT contrast agent, providing accurate guidance for tumor treatment. This research showed that the designed multifunctional nanosystem possessed high hydrogen-loading capacity, realizing selective PTT/hydrogen therapy while using the US/CT dual-peak imaging function to accurately guide the antitumor therapy. This multifunctional and multiple treatment strategy provided a theoretical basis for future clinical transformation.

**Fig. 7:**
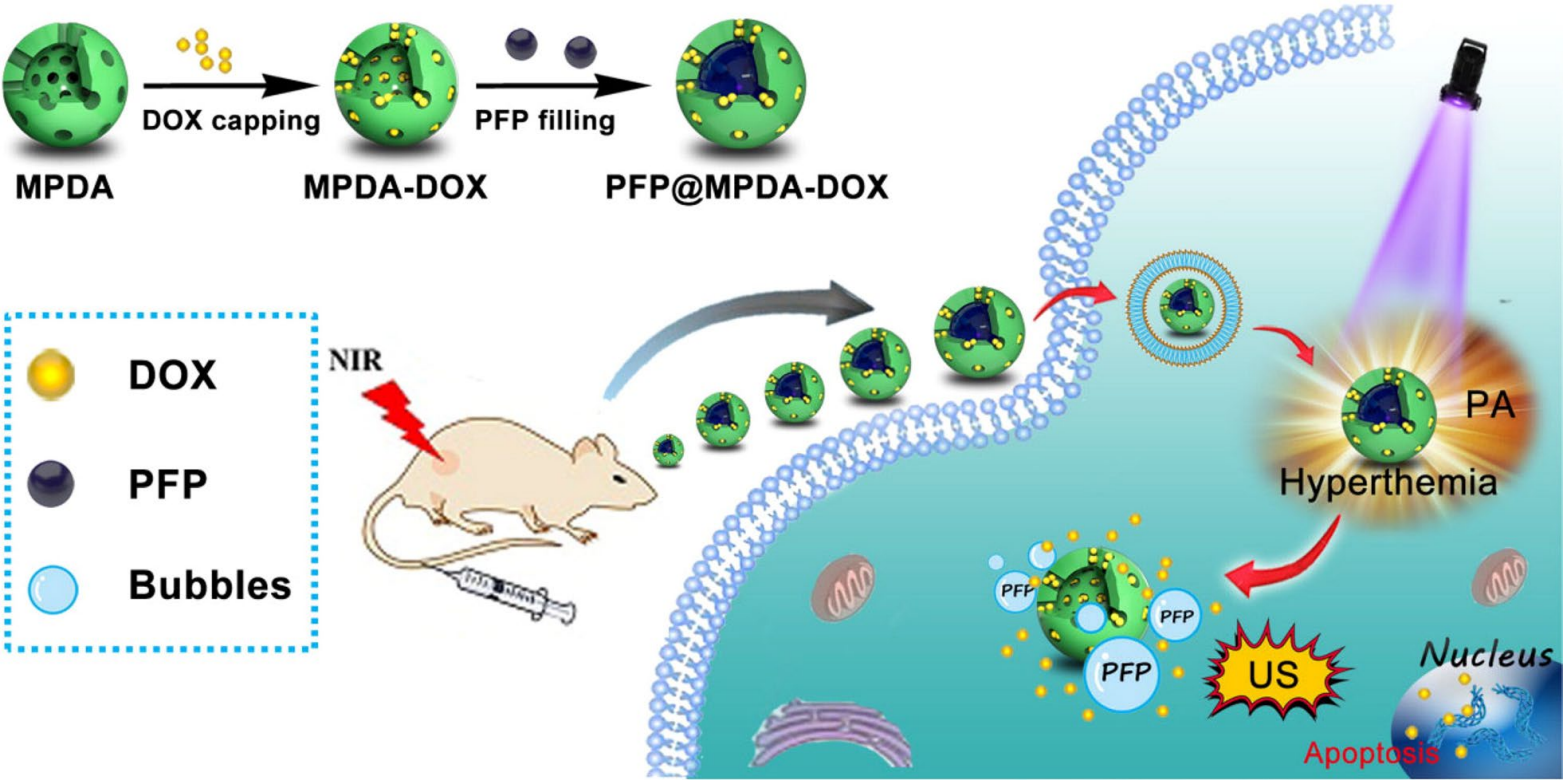
Schematic illustration of the procedure used to fabricate PFP@MPDA-DOX nanotheranostics for PA/US guided chemo-photothermal therapy of tumor. Reprinted (adapted) with permission from Yang et al. [129]. (Copyright © 2021Yang et al. Drug Delivery published by Taylor & Francis)

Photoacoustic (PA) imaging is a non-destructive testing technology that has developed rapidly in recent years. Owing to its high resolution and high contrast characteristics, it has become the main direction of biomedical testing technology development [126–128]. PA imaging also has a wide range of applications in MPDA-based platforms. Yang et al. [129] designed a multifunctional cancer theranostics nano-platform. The PFP@MPDA-DOX nano-platform exhibited significant NIR absorption and high photothermal conversion efficiency (η = 45.6%), and can be used as a multifunctional nanosystem for PA imaging and photothermal therapy (Fig. 7). First, MPDA NPs were synthesized through the nanoemulsion assembly method. The surface area of the MPDA NPs was 51.3 m^2^ g^−1^, and the pore size was 7.44 nm. MPDA was used as a carrier to encapsulate hydrophobic perfluoropentane (PFP), and DOX was fixed on the surface of MPDA through Schiff base reaction, affording PFP@MPDA-DOX. When PFP@MPDA-DOX was irradiated by a NIR laser, mild hyperthermia induced the liquid-gas phase transition of PFP. The bubbles not only promoted the uptake of chemotherapeutics by tumor cells but also could be used as a contrast agent for US imaging. Research showed that the PFP@MPDA-DOX nano-platform had great potential in US/PA dual-mode imaging. The developed MPDA-based nano-platform has great potential in cancer nanotherapy.

Gas therapy has emerged as a cancer treatment strategy with broad application prospects. Relevant studies have shown that certain gas signal molecules in the human body, including nitric oxide (NO), carbon monoxide (CO), and hydrogen sulfide (H_2_S), exerted unique therapeutic effects against cancer, inflammation, and cardiovascular diseases [130–132]. Recently, it was discovered that CO, an endogenous gas molecule, was able to kill cancer cells without affecting normal cells, showing a significant anticancer effect. However, the uncontrollable release and accumulation of CO gas can lead to poisoning risks. In addition, the reported CO gas therapy has a limited drug-loading efficiency and cannot reach an effective concentration to treat tumors. Therefore, constructing a nanocarrier delivery system with high drug-loading capacity, stimulus responsiveness, and imaging guidance functions is crucial for precise gas therapy. Wu et al. [133] reported a new type of MPDA nano-diagnostic/therapeutic agent (MnCO@MPDA NPs), which exerted MRI/PA imaging functions and targeted gas transport, thus successfully realizing medical imaging-guided noninvasive cancer treatment. The prepared MPDA NPs (particle size, approximately 200 nm) were used to efficiently load the gas prodrug manganese carbonyl. The material showed high light-to-heat conversion efficiency (η = 40%) and good biocompatibility, with a drug-loading capacity of 60.3%. Manganese carbonyl (MnCO), a Fenton-like reagent, could respond to the high content of H_2_O_2_ and H^+^ in the TME. Fenton-like reaction occurred to produce CO gas and Mn^2+^ (MRI T_1_ contrast agent). The agent exerted potent cytotoxicity only to tumor cells overexpressing H_2_O_2_, and caused almost no cytotoxicity to normal cells, thus exhibiting highly selective anticancer effects and good biological safety. The nano-platform system monitored the distribution of nanoparticles and the treatment process through MRI/PA imaging. The CO gas released at the tumor site promoted mitochondrial synthesis and enhanced reactive oxygen species to induce cancer apoptosis while exerting a synergistic anticancer effect with PTT. In animal experiments, the tumor ablation effect was significant. This study established a solid empiric rationale for the development of nano-gas diagnostic/therapeutic agents and medical imaging-guided tumor treatment technologies. It has crucial research significance and promising application value.

Multispectral optoacoustic tomography (MSOT) is a new imaging technique for tumors. It combines the benefits of optical and US imaging. In this method, pulsed laser is used as the light source, and the image reflects the difference in the light absorption distribution of various substances in biological tissues; thus, it inherits the advantages of optical imaging in terms of functionality and sensitivity. Moreover, MSOT detects external ultrasound signals, benefiting from the advantages of US in imaging depth and resolution. Therefore, MSOT has broad application prospects and far-reaching technological innovation significance in the field of biomedical research [134–137]. The MPDA-WS_2_@MnO_2_ nanoparticles prepared by Wang et al. [138] applied three-modal contrast agent (CT/MSOT/MR) imaging to provide real-time guidance and monitoring during cancer theranostics. In this study, MPDA NSs were used as MSOT probes to monitor PTT in real-time. Tungsten disulfide quantum dots (WS_2_ QDs) were embedded in MPDA nanoparticles (MPDA NSs) as radiosensitizers and CT contrast agents. Manganese dioxide (MnO_2_) reacted with H^+^/H_2_O_2_ and then released Mn^2+^ to activate MRI. The combination of CT/MSOT/MR images enhances the potential of obtaining more accurate spatial and physiological visual data, which is beneficial for guiding precision combinational treatment and real-time monitoring in cancer theranostics. It opens new horizons for combinational multimodal imaging.

Single-photon emission computed tomography-computed tomography (SPECT-CT) is a combination of high-end SPECT and multi-slice spiral CT into an integrated device, that is, a single photon emission CT plus X-ray CT scanner. In addition to providing SPECT functional information, it can also provide CT anatomical information. Precise registration and same-machine fusion further enhance the accuracy of disease diagnosis. Through tomographic image fusion technology, SPECT images can be accurately positioned on CT images. The CT image and the anatomical image are then superimposed and overlapped, realizing the complementary advantages of SPECT and CT [139, 140]. This instrument plays an important role in the early detection, diagnosis, and treatment of bone tumor metastasis, thyroid disease, urinary system disease, digestive system disease, and circulatory system diseases. Huang et al. [141] prepared an MPDA nanodrug delivery system and modified its morphology through component-adjusting Pluronic micelle-guided polymerization. MPDA was used not only as a nanocarrier to radiolabel ^131^I but also as a photothermal conversion agent for PTT to promote radiotherapy. The study found that MPDA NPs with a cerebroid pore channel structure (CPDA) showed the highest iodine-carrying capacity and a high photothermal conversion efficiency owing to their maximum specific surface area and unique morphology. The SPETC/CT molecular imaging technology could accurately guide the clinical treatment of anaplastic thyroid carcinoma.

MPDA nanoformulations are also used to overcome multidrug resistance [142], intratumoral hypoxia [143], and the fluorescence quenching characteristics of drugs [144]. At present, a MPDA nanotherapy system is combined with hyperthermia therapy, chemotherapy, and multimodal imaging-guided therapy to control drug resistance in tumor cells. With the development of MPDA as a tumor treatment, research on photothermal conversion-chemical drug synergistic therapy and photothermal conversion-gene drug synergistic therapy can provide a theoretical basis for the clinical development of new nanoformulations.

## Discussion

In the past few decades, researchers had been devoted to developing new cancer treatments. Research on PDA and nanomaterials has played a crucial role in cancer treatment. The emergence of MPDA has pioneered a new field of cancer research. This review summarizes several applications of MPDA in cancer treatment. MPDA, a novel mesoporous organic polymer, has mesoporous silicon material structural features and diverse polymer modifiable and functional properties. It also possesses excellent optical and electrical properties. Thus, MPDA has promising application potential and research significance in tumor treatment and the entire field of nanomedicine. In the past years, great advances have been achieved in MPDA-based drug delivery systems. Nevertheless, translating these novel findings into clinical practice is still difficult. Firstly, the structures, degradability, and pharmacokinetics of MPDA materials should be systematically investigated. Secondly, essential information regarding the blood circulation properties, such as clearance time in the body and possible immunogenicity and accumulation in tissues, should be obtained before the clinical translation of MPDA. Thirdly, MPDA is a new class of materials obtained by modifying the preparation method of polydopamine. In the future, researchers should carefully explore the toxicity of MPDA materials before aiming for wide applications because even though many reports have shown that MPDA materials exhibit excellent biocompatibility, it has also been reported that PDA can cause unwanted side effects, which can affect their important applications, especially in biomedicine and catalysis [145]. In addition, in accordance with the ethical guideline for clinical application, novel treatment methods must be tested in humans; thus, their effectiveness and safety must first be proved. With the development of nanotechnology, the potential application of MPDA nanomaterials in clinical cancer treatment will increase.

## Data Availability

Not applicable.
